# Molecular insights into polyurethane biodegradation in *Pseudomonas protegens*

**DOI:** 10.1128/mbio.03544-25

**Published:** 2026-01-20

**Authors:** Lucie Semenec, Ram Maharjan, Vaheesan Rajabal, Aidan P. Tay, Xin Xu, Hannah Lott, Fiona S.B. Facey, Hue Dinh, Sasha G. Tetu, Thomas C. Williams, Ian T. Paulsen, Amy K. Cain

**Affiliations:** 1 ARC Centre of Excellence in Synthetic Biology, Department of Natural Sciences, Macquarie University 7788, Sydney, New South Wales, Australia; National Institutes of Health, Bethesda, Maryland, USA

**Keywords:** *gacS*, functional genomics, oxidative stress, plastic degradation, Fenton reaction, pyoverdine

## Abstract

Global accumulation of difficult-to-recycle plastics, including polyurethane (PU), poses a significant environmental threat, as current recycling strategies are insufficient to meet the growing demand. *Pseudomonas protegens* is capable of degrading PU and may offer a promising nature-inspired solution. However, the genetic and regulatory mechanisms underlying microbial PU biodegradation remain poorly understood. To address this, we employed transposon insertion sequencing (TIS), a high-throughput functional genomics approach, to identify genes essential for PU degradation under plastic exposure conditions. A transposon mutant library of *P. protegens* Pf-5 was enriched on two types of PU: a polyester-polyurethane dispersion (Impranil DLN) and a thermoplastic polyurethane (Avalon AE). Through this approach, we identified a key global regulator encoding a sensor kinase, *gacS,* that plays an inhibitory role in PU degradation, so that *P. protegens* Pf-5 lacking *gacS* displayed enhanced capacity to degrade PU. Transcriptomic and phenotypic analyses revealed that disruption of *gacS* resulted in increased activity of siderophore-mediated iron acquisition, while negatively impacting sulfur homeostasis and oxidative stress responses. We propose that this imbalance in iron homeostasis and oxidative stress in the *gacS* mutant triggers a Fenton reaction, leading to the accumulation of reactive oxygen species, which in turn enhances PU degradation via oxidative processes. These findings establish that siderophores involved in iron acquisition, such as pyoverdine, could act as catalysts for plastic degradation. Our results not only provide a deeper understanding of the genetic and regulatory basis of PU biodegradation but also open new avenues for leveraging bacterial pathways and siderophores for innovative plastic recycling strategies.

**IMPORTANCE:**

The polyurethane (PU) market is projected to reach $94 billion USD by 2028 and spans many industries, including automotive, construction, medical, furniture, and fashion. However, less than 30% of PU plastics are recycled, with the remainder going to landfill. The inherent recalcitrant nature of PU makes it challenging to study naturally occurring routes of PU degradation, like microbial biodegradation and its underlying genetic mechanisms. To address this, we developed a high-throughput screening method using transposon-directed insertion site sequencing (TraDIS) on the known PU-degrading strain *Pseudomonas protegens* Pf-5. This approach identified a key global regulatory gene (GacS), the mutant of which acts as a “hyperdegrader” with a faster PU degradation rate. Subsequent transcriptomic and phenotypic analyses revealed an unsuspected PU-degradation mechanism involving a siderophore-mediated Fenton reaction. These findings highlight the importance of using high-throughput functional genomics to uncover novel genes and pathways involved in plastic biodegradation. Our findings not only advance the understanding of PU degradation but also open new avenues for developing innovative solutions for plastic waste management.

## INTRODUCTION

Of the 350–400 million tonnes of plastics produced yearly, polyurethane (PU) is the fifth most abundantly produced plastic type and is used in numerous products like foams, synthetic leather, coatings, sealants, and adhesives (1). This polymer is made from the chemical reaction of diisocyanates with polyols, which form a urethane linkage; however, various other moieties can be added, like urea, aromatic groups, esters, and ethers (2). Traditional processing of PU waste is more challenging than other plastic types due to its variable composition, often containing flame retardants (3). Thus, landfilling and incineration are not performed with PU, and instead, recycling is the only viable route for dealing with PU waste. Although chemical recycling is currently used, it requires high temperatures, which release noxious fumes, making it an imperfect solution. Alternatively, various microorganisms can biodegrade PU, providing a promising natural repository of useful degradation enzymes. Both fungal and bacterial species have been shown to biodegrade PU, including *Aspergillus* sp. (4), *Cladosporium halotolerans* (5), *Embarria clematidis* (6), *Alicycliphilus denitrificans* (7), *Rhodococcus* sp. (8), *Trichoderma* sp. (9), *Pestalotiopsis microspore* (10), *Exophiala jeanselmei* (11), *Delftia acidovorans* (12), *Curvularia senegalensis* (13), and various *Pseudomonas* sp. (14, 15), the latter of which is most commonly reported on. Interestingly, each microbial species has a different repertoire of enzymes it utilizes for biodegradation, including ureases, lipases, esterases, hydrolases, proteases, and decarboxylases, which belong to either the hydrolase or lyase class of enzymes. Although many studies have investigated the enzymes involved in microbial PU degradation, much less is known about the regulatory pathways involved.

To gain a deeper understanding of this complex metabolism, it is important to apply a system-wide approach using functional genomics; however, less than a handful of studies have done this to date. In one study of *C. halotolerans*, RNA-seq was performed on the fungal strain grown on colloidal polyester PU dispersion (PUD), Impranil DLN (Impranil), and compared with growth on glucose as the sole carbon source (5). The authors found an enrichment in ester reductase, lipase, and peroxidase enzymes, which were significantly induced within 24 h of growth on Impranil, indicating that PU may signal induction of their expression. In another functional genomics study, authors investigated the RNA-seq profile of *Pseudomonas capeferrum* TDA1 grown on a PU precursor, 2,4-toluene diamine. They found that genes involved in environmental stress response, including metal ion stress, protein misfolding, and transporters, were significantly overexpressed (16), indicating the breakdown products of PU have some level of toxicity even though they can be utilized as carbon sources. A previous study by Hung et al. (17) discovered a link between carbon source utilization and PU degradation in *Pseudomonas protegens* Pf-5, where carbon catabolite repression (CCR) during growth on glucose and Impranil inhibited the ability of the strain to degrade Impranil. Conversely, when citrate was provided instead as a carbon source, PU degradation was enhanced. Interestingly, expression of genes *pueA/B*, encoding PueA/B polyurethanase enzymes, increased in the presence of citrate and Impranil, suggesting regulation by CCR (17). Although it is known that CCR is regulated by the CbrA/B signal transduction pathway in *Pseudomonas* spp. (18), there is no confirmed link between this pathway and PU degradation.

The use of transposon insertion sequencing (TIS) is a powerful functional genomics approach for probing the importance of every gene in the genome in providing fitness in any selective condition. However, this method has not yet been applied to identify key genes involved in biodegradation. In this study, we employed the TIS approach of transposon directed insertion site sequencing (TraDIS) (19) to uncover genes and pathways associated with bacterial PU degradation by *P. protegens* Pf-5. *Pseudomonas* spp. are the most studied PU-degrading bacteria (20). Transposon mutant libraries were screened on PUD, Impranil, and thermoplastic polyurethane (TPU), Avalon 80 AE (Avalon 80), to compare the pathways involved in the biodegradation of different PU types. Our data identified novel genes and pathways not previously linked to PU degradation, for both PUD and TPU. Using the Biocyc and KEGG databases, the genes were mapped onto functional pathways, highlighting several important to PU degradation. Notably, GacS, a sensor histidine kinase of the GacA/S two-component regulatory system, exhibited increased transposon insertions in TPU conditions versus media containing only citrate as a carbon source. To further investigate the novel role of GacS in PU degradation, we conducted transcriptomics analysis of the Δ*gacS* mutant. This revealed that disruption of GacS led to the dysregulation of iron and sulfur homeostasis and oxidative stress responses. Subsequent phenotypic analysis demonstrated that the *gacS* mutant utilizes a siderophore-mediated Fenton reaction to generate reactive oxygen species, which ultimately contribute to the PU breakdown. Overall, this work identifies the first major regulator of PU degradation and will help in the generation of engineered strains with enhanced plastic degradation capabilities in the future.

## RESULTS AND DISCUSSION

### Defining conditions for PUD and TPU mutant library selection

To identify the genes or pathways important in *P. protegens* Pf-5 for PU degradation, we employed a fitness-based functional genomics technique, TraDIS (19, 21). A previous study showed that *P. protegens* Pf-5 is able to degrade PU optimally in M9 citrate-agar medium when citrate is provided as an additional carbon source (17). Therefore, we used the M9 citrate-agar medium to perform TraDIS experiments with two different PU materials: Impranil (a polyurethane dispersion, PUD) and Avalon (a thermoplastic polyurethane, TPU). Although the exact structure of Impranil is not fully known, it is reported to consist of a polyhexane/neopentyl adipate polyester and hexamethylene diisocyanate (22), and its putative structure has been described (12, 23). The structure and composition of Avalon 80 are also not fully known; however, it is best described as a polyester-based TPU film. In general, TPUs are more resistant to degradation than PUDs and, as such, generate less microplastic pollution, making for an important comparison with PUD.

**Fig 1:**
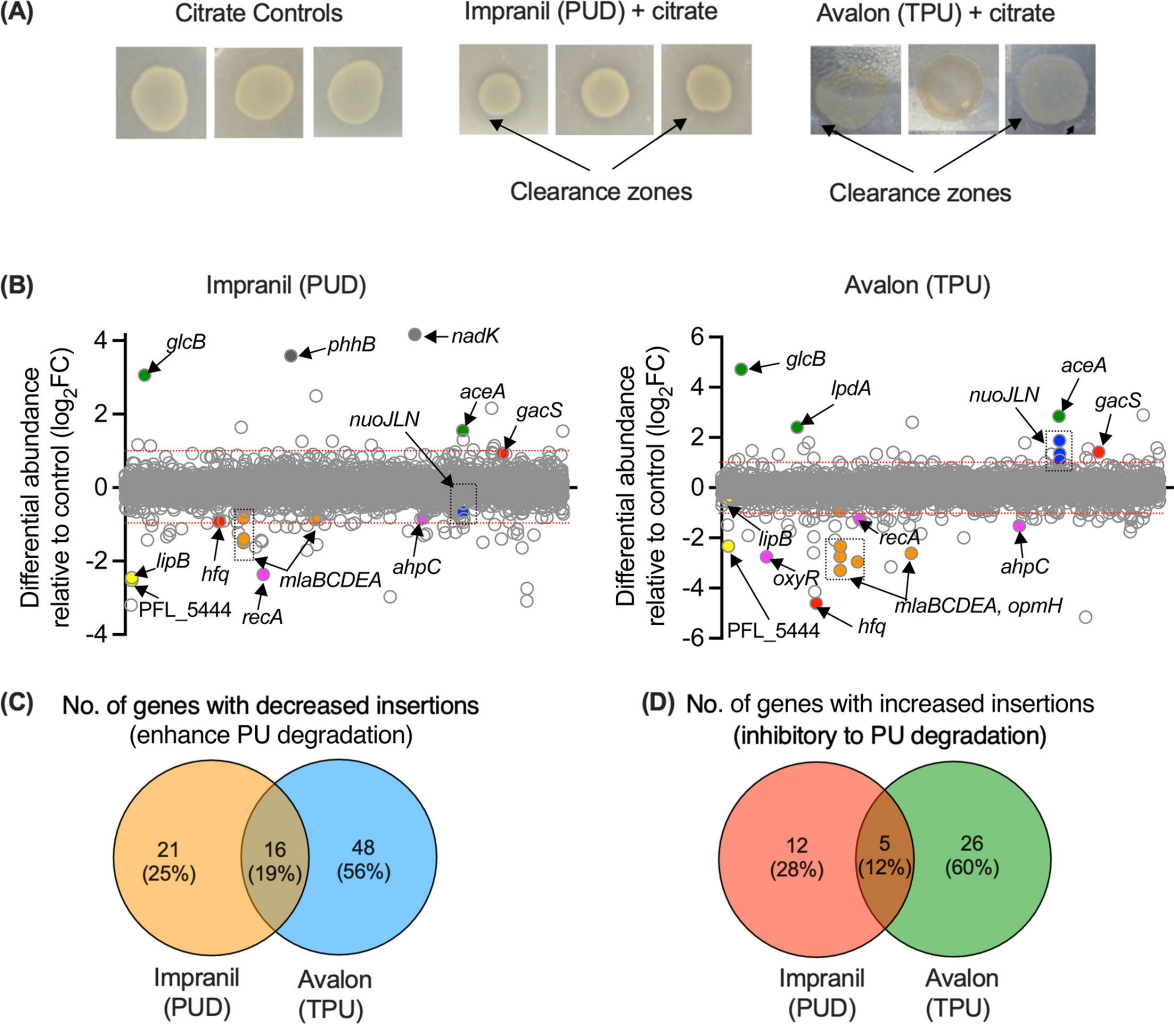
TraDIS experiment on PU with *P. protegens* Pf-5. (**A**) M9 minimal media agar plates containing citrate only (control), Impranil DLN + citrate (test), and Avolon 80 + citrate (test). Black arrows indicate clearance zones corresponding to PU degradation on Impranil- and Avolon-citrate plates. For Avalon-citrate agar plates, clearance zones were visualized by using oblique lighting. (**B**) The effect of 3 g/L Impranil (left panel) and 3g/L Avalon (right panel) on differential transposon insertion frequency (differential abundance) between the control and test PU agar plates as measured by log2-fold change (log2FC). Each gene is positioned along the genome according to its gene start position. Each circle represents a gene, and genes within the red dotted lines have non-significant log2FC values log2FC ≥ 1.0, *P* value ≥ 0.05. Significant genes (log2FC ≥ 1.0, *P* value ≤ 0.05) for those above the red dotted line or below are colored based on known function. (**C**) Venn diagram of genes with a significant decrease in insertions (log2FC > 1.0, *P* value ≤ 0.05) between citrate only vs citrate + Impranil (PUD) or citrate + Avalon (TPU). (**D**) Venn diagram of genes with a significant increase in insertions (log2FC < 1.0, *P* value ≤ 0.05) between citrate only vs citrate + Impranil (PUD) or citrate + Avalon (TPU).

PU degradation was evident on Impranil citrate agar plates after 3 days of growth with a distinct zone of clearance (Fig. 1A; Fig. S1). However, due to the transparent property of Avalon 80 and its greater resistance to degradation, significant but smaller zones of clearance were present, which required oblique lighting for clear visualization (Fig. 1A). We then harvested, extracted DNA, and sequenced the transposon mutant libraries grown with the two types of PU, and identified genes with significant differential insertions between PUD or TPU conditions compared to the no plastic control (M9 + citrate). Non-essential genes with an increased mutant abundance in the presence of PUs, relative to the citrate control, were considered to have a role in preventing PU degradation (Fig. 1B). Conversely, genes whose mutants decreased in abundance were considered to have a role in enhancing PU degradation.

We identified 37 and 64 genes with significantly decreased transposon insertion abundance in PUD and TPU conditions, respectively, compared to controls using standard cut-offs (log2FC > 1 and *P* < 0.05), indicating a potentially important role in facilitating *P. protegens* Pf-5’s ability to degrade PU (Fig. 1B and C). Of these genes, 16 (19%) were common to both PUD and TPU conditions (Tables S1 and S2). Similarly, 17 and 31 genes showed significantly increased abundance relative to the control in PUD and TPU conditions, respectively, with 5 (12%) genes common to both PUs (Fig. 1D), suggesting their potential role in inhibiting PU degradation. As a validation of our TraDIS functional screening, the known PU-degrading gene *lipB* (D3X12_00540)*,* encoding lipase B (17, 24), was identified among the mutants with decreased abundance in PUD (Fig. 1B; Table S1).

### Membrane lipid asymmetry is important for fitness on TPU and PUD

**Fig 2:**
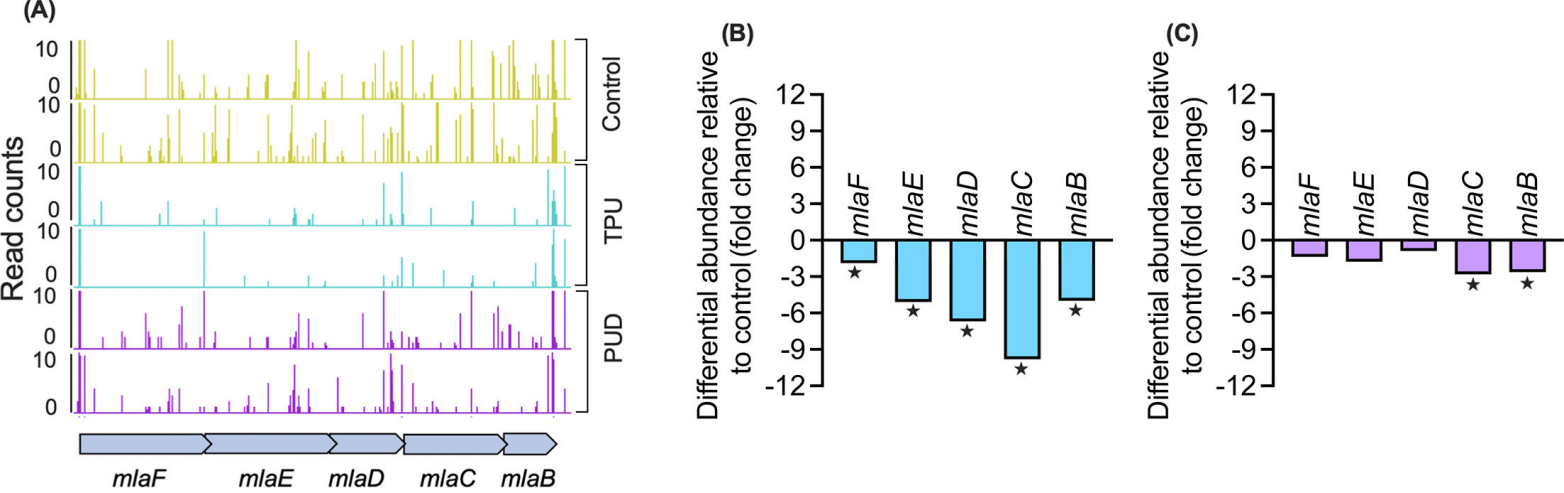
(**A**) Mla pathway genes with significantly lower insertions in Avalon TPU and PUD conditions as visualized using the Artemis genome viewer with window size set to 1 and max/min = 10. Insertion mutations in genes of the mla transporter locus in controls top two layers, TPU (Avalon) middle two layers, and PUD bottom two layers. (**B, C**) Differential abundance of Mla genes in TPU (**B**) and PUD (**C**).

The membrane lipid asymmetry (Mla) pathway genes *mlaBCDEF* encode proteins that include an ABC transporter substrate-binding protein, outer membrane lipid asymmetry maintenance protein, permease, and phospholipid transporter, and belong to the Membrane Cell Entry (MCE) family (25–27). These genes showed significantly reduced insertions in TPU (and *mlaBC* in PUD) compared to citrate control (Fig. 2).

In *Escherichia coli*, the porin OmpC interacts with MlaA, but its role in the system remains elusive (25). Interestingly, while *P. protegens* Pf-5 lacks an *ompC* gene, the *ompH* gene displayed a 7.5-fold decrease in insertion in TPU conditions, indicating it may play a similar role in *P. protegens* that OmpC plays in *E. coli*. The Mla system maintains outer membrane phospholipid asymmetry by actively transporting phospholipids between membranes, facilitating hydrophobic molecule transport across the hydrophilic periplasm (26).

PU degradation generates short-chain fatty acids (e.g., adipic acid) and long-chain alkanes (e.g., hexadecane) (5, 12). While shorter fatty acids can diffuse or enter via porins, long-chain (>C12) molecules require specialized import (28). In *E. coli*, this involves FadD and FadL (29), but we saw no significant changes in these genes in either PU condition, suggesting alternative import routes. The MCE system in *Mycobacterium* is involved in both lipid asymmetry and fatty acid import (25, 30), raising the possibility that the Mla pathway in *P. protegens* Pf-5 also imports lipophilic PU breakdown products. The absence of other significant porins/permeases further supports this.

Fatty acid import typically downregulates biosynthesis, but we found no significant insertion differences in fatty acid biosynthesis genes. Fatty acid degradation genes *fadB* and *fadA* had 2.8- and 1.7-fold lower insertions under TPU. Five genes important in both PU types encode uncharacterized proteins, representing potential novel PU degradation genes. Genes encoding a carboxypeptidase (PFL_5444) and a putative hydrolase (PFL_2023) also showed reduced insertions in both conditions, suggesting a role in processing imported PU breakdown products.

### Glyoxylate shunt and the electron transport chain are deleterious for PU degradation

Among the five genes with increased insertions (negative fitness effects) shared between PUD and TPU were *glcB* and *aceA,* which encode malate synthase (GlcB) and isocitrate lyase (AceA), respectively (Fig. 1B). GlcB and AceA are key components of the glyoxylate shunt (GS), which bypasses the decarboxylation steps of the tricarboxylic acid (TCA) cycle and more efficiently generates oxaloacetate for gluconeogenesis and biomass production (31). The GS is essential for growth on acetate, fatty acids, and alkanes as a sole carbon source in various bacterial species, such as *Corynebacterium* sp. and *Acinetobacter oleivorans* (32). However, in *P. aeruginosa*, where glyoxylate shunt enzymes were inactive during growth on alkanes (33). This latter finding agrees well with our results, where glyoxylate shunt genes appear to reduce the fitness of *P. protegens* Pf-5 growth on media containing TPU or PUD. Additionally, in *P. aeruginosa,* the presence of gluconeogenic precursors, oxaloacetate and pyruvate, shifts flux away from the GS and toward the TCA cycle by inhibiting isocitrate lyase, GlcB (34). As *glcB* mutants were enriched in both PU types, PU breakdown may yield metabolites that inhibit GS or generate alternative carbon/electron sources, bypassing GS needs.

We also observed increased insertions in electron transport chain (ETC) complex I subunit genes (*nuoJ*, *nuoL*, *nuoM*, *nuoN*) under TPU (Fig. 1B). In PUD, these genes were not significant, but loss of *nadK* (NAD kinase) reduced fitness (log2FC = 4.2, *P* < 0.001). The ETC complex I plays an important role in oxidative stress protection in Pseudomonas species (35). This is because reactive oxygen species (ROS) are primarily produced when electrons leak from ETC complexes I and III and reduce O_2_ and generate O_2_^−^ (35, 36). This suggests that the ETC may also play a role in impeding PU degradation, potentially through reducing ROS generation. Interestingly, like the *nuo* cluster, NAD+ is essential for cellular redox balance and ADP-ribosylation of DNA, which further aids in DNA repair (37, 38).

The GS is also linked to oxidative stress (39), and thus interplay between the GS and ETC in the context of PU degradation highlights the metabolic challenges posed by oxidative stress. It has been shown that the generation of alternate electron acceptors, such as CO_2_, during PU degradation (40) may explain this connection. Interestingly, the ETC is downregulated in nonalcoholic fatty acid liver disease, where excess ROS are generated from increased fatty acid oxidation (41). Given the similarity between PU and fatty acids, PU breakdown may also generate ROS. This could explain why PU conditions are enriched for transposon insertions within the ETC machinery, as ROS production may disrupt ETC function and create selective pressure against its activity.

### Genes involved in oxidative stress were depleted under PU conditions

Under TPU, we found decreased insertions in oxidative stress response genes, notably *oxyR* (log2FC = –5.1, *P* < 2E–37), a regulator controlling *ahpC–ahpF*. Genes for lipopolysaccharide biosynthesis and biofilm formation (*algC*, *algG*, *pslF*, *pslH*) (42, 43) showed significantly fewer transposon insertions (Table S1), suggesting their importance in mitigating ROS-driven membrane damage during oxidative stress (44). A peroxiredoxin encoding gene, *ahpC,* also exhibited significantly fewer insertions (log2FC −1.5) in TPU-grown libraries, further supporting the generation of ROS during TPU biodegradation.

Interestingly, in *P. aeruginosa*, the *oxyR* gene is located within the same operon as *recG*, which encodes a DNA repair enzyme (37, 45). This genomic arrangement appears to be conserved in *P. protegens* Pf-5, suggesting a coordinated response to oxidative stress that involves both ROS detoxification and DNA repair mechanisms. Together, these findings underscore the complex interplay between metabolic pathways like the glyoxylate shunt, oxidative stress responses, and membrane integrity maintenance during PU degradation, highlighting the adaptive strategies employed by bacteria to cope with the metabolic and oxidative challenges posed by PU breakdown.

### Global regulators, GacS and Hfq, have opposite roles during growth on TPU and PUD

Our TraDIS functional genomics identified two global regulators, GacS and Hfq, that potentially play a role in PU degradation. GacS is a sensor kinase encoded by *gacS*, which is a part of the two-component global regulatory GacS/A system (46). Hfq, a small RNA chaperone protein widely present in prokaryotes, plays an important global regulatory role in bacterial physiology and metabolism (47). The GacS/A system relies on small RNAs, such as RsmXYZ-RsmE, to regulate various metabolic pathways, including the synthesis of enzymes, such as alkaline protease, lipases, and pyocyanin cyanide (48, 49). Since Hfq acts as a chaperone for small RNAs, and the GacS/A system depends on them for regulation, we initially expected *gacS* and *hfq* to have similar effects during TraDIS enrichment. Surprisingly, our results showed opposing roles: *gacS* mutants were enriched (FC = 2.7; *P* < 0.005) under TPU conditions, while *hfq* mutants were depleted (FC = −24.4, *P* < 0.0002). This indicates that GacS negatively regulates PU degradation, while Hfq is essential for the process.

The GacS/A system post-transcriptionally modulates multiple pathways, including those involved in extracellular enzyme production, biofilm production, carbon catabolite repression, the Mla pathway, and oxidative stress responses (50–52). Given its broad regulatory role, GacS may serve as a key master regulator of physiology and metabolic processes involved in PU degradation. Therefore, we focused on GacS, further investigating its role in PU degradation.

### Validation of the negative role of GacS in PU degradation

Given the wide range of pathways controlled by the GacS/GacA two-component global regulatory system, we aimed to determine how GacS would indeed impede PU degradation. To investigate this, we constructed a *gacS* deletion mutant in *P. protegens* Pf-5 using the pCasPA/pACRISPR and *λ*-Red recombination system (53). As a control, we also generated a *pueA* mutant, a polyurethanase gene essential for PU degradation, since PU degradation has been previously reported to be significantly diminished in Δ*pueA* deletion mutants (17, 54).

**Fig 3:**
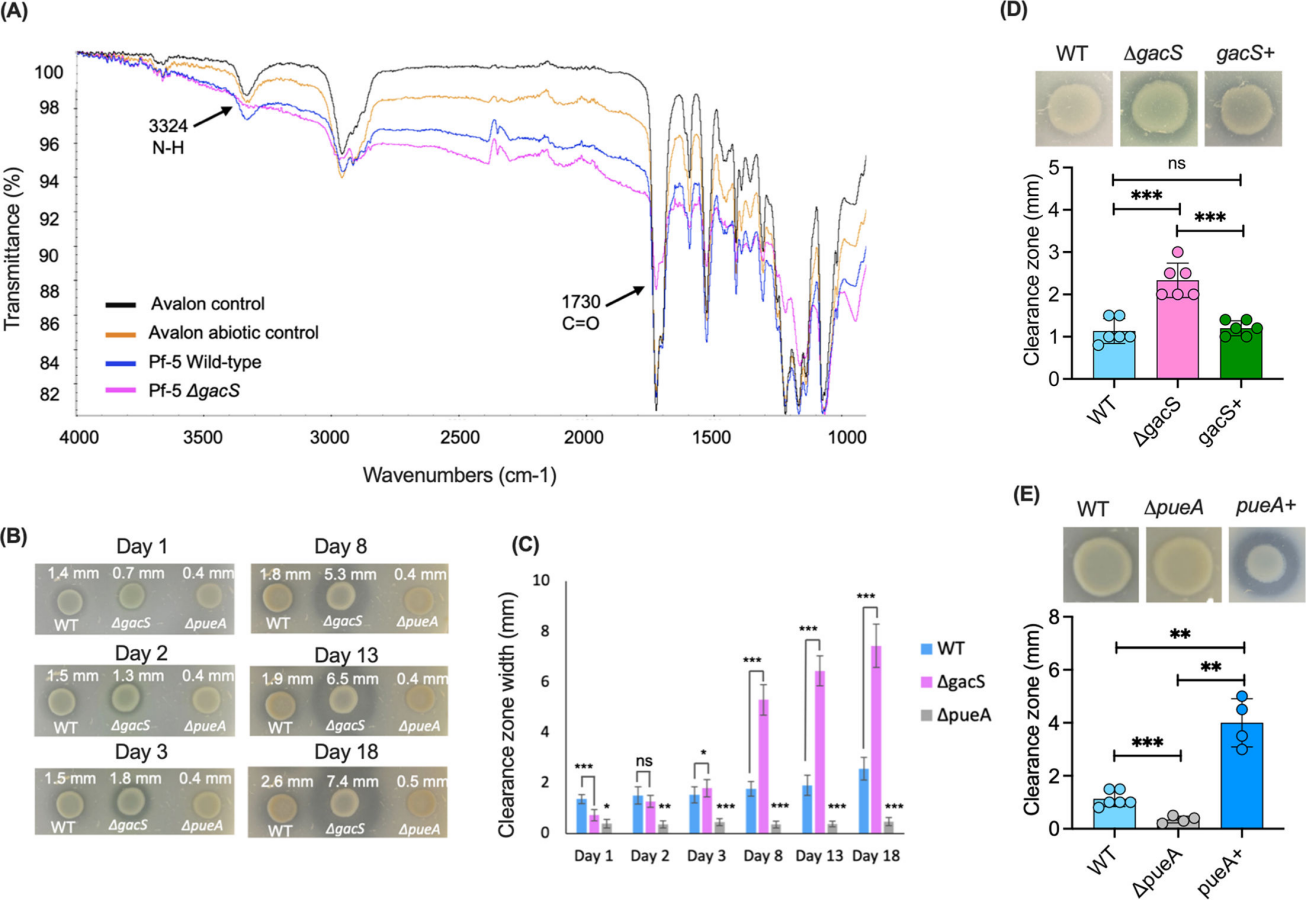
Polyurethane degradation ability of *P. protegens* Pf-5 wild-type (WT), Δ*gacS,* and Δ*pueA* mutant strains. (**A**) Degradation of Avalon 80 (TPU) by *P. protegens* Pf-5 wild-type and its Δ*gacS* mutant as measured by FTIR spectroscopy. Avalon control (Avalon only, to assess baseline spectra), Avalon abiotic control (Avalon under the same incubation conditions but without cells, to confirm that degradation is not due to abiotic factors), and Pf-5 WT and Δ*gacS* (Avalon treated with the wild-type and its Δ*gacS* mutant for PU degradation). (**B**) Impranil clearance zones for *P. protegens* Pf-5 WT, Δ*gacS,* and Δ*pueA* mutant strains. (**C**) Clearance zone widths were measured by ImageJ and are presented as the means ± standard deviations from three biological replicates. (**D and E**) Impranil clearance zones for *P. protegens* Pf-5 WT, Δ*gacS,* and Δ*pueA* mutant strains and their respective complemented strains at day 3. Significance levels were calculated with a two-tailed Student's *t*-test from at least three biological replicates (ns = not significant, *P < 0.05, **P < 0.01, ***P < 0.001).

To assess the degradation activity of *P. protegens* Pf-5 and its Δ*gacS* mutant, we first examined TPU Avalon 80 degradation using Fourier-transform infrared (FTIR) spectroscopy. Molten TPU was drop-cast onto glass slides and exposed to culture filtrates of *P. protegens* Pf-5 wild-type and the Δ*gacS* mutant, compared to previously established characteristic markers of PU breakdown were examined (12, 55). The FTIR spectra revealed a decrease in the nitrogen N–H stretch (3,324 cm^−1^) of urea/urethane groups and the carbonyl C=O stretch (1,730 cm^−1^), corresponding to ester/urethane groups in Avalon 80 (Fig. 3A) for the Δ*gacS* mutant. This confirmed that Δ*gacS* exhibited enhanced TPU degradation compared to the wild-type strain.

Although we did not observe a statistically significant difference in transposon insertions in the *gacS* gene when mutant libraries were grown on Impranil (FC = 1.4, *P* = 0.37), we nonetheless investigated whether the Δ*gacS* mutant affected Impranil degradation. Since Impranil clearance is most easily observed on Impranil + citrate agar plates, we used this method for initial screening.

After 1 day of growth on Impranil + citrate agar, the Δ*gacS* mutant had a significantly smaller clearance zone (0.73 ± 0.08 mm) compared to (1.37 ± 0.02 mm) of the *P. protegens* Pf-5 wild-type strain, whereas the Δ*pueA* mutant showed only (0.39 ± 0.11 mm) clearance (Fig. 3B and C; Table S3). By day 3, the clearance zone observed for the Δ*gacS* mutant started to exceed that of the wild-type strain (Fig. 3B). By day 8, clearance (5.3 ± 0.58 mm) was 171% higher in the Δ*gacS* mutant compared to the Δ*pueA* mutant and 97% higher than the clearance of 1.8 ± 0.07 mm of wild-type Pf-5 strain (Table S3). Since we collected the DNA for TraDIS at day 3, when Δ*gacS* (1.8 ± 0.14 mm) and wild type (1.5 ± 0.08 mm) exhibited similar degradation rates, it is possible that *gacS* becomes more important to fitness at later stages of growth on Impranil degradation. The significant difference in clearance between the wild-type and the Δ*gacS* strain suggests that the Δ*gacS* mutant acts as a PU hyperdegrader.

To demonstrate that the observed phenotypes for the Δ*gacS* and Δ*pueA* were not due to polar effects of gene deletions, we complemented each mutant with its corresponding wild-type genes. As shown in Fig. 3D and E, the complemented strains restored PU degradation phenotypes to near-wild-type levels, confirming that the observed effects are specific to the deletion of *gacS* and *pueA* and were not due to polar effects. Interestingly, the *pueA*-complemented strain (*pueA*^+^) showed a significantly higher level of clearance, likely due to increased expression compared to the wild type.

### Transcriptomic analysis to define GacS-dependent PU degradation mechanisms

To identify the molecular mechanisms underlying PU biodegradation in *P. protegens* Pf-5, we conducted comparative transcriptomics by RNA-sequencing (RNA-seq) on both the Δ*gacS* mutant and the WT strain during exposure to Impranil PU (Fig. S2). Comparative analysis of a total of 6,401 genes revealed that 21% (1,333) of the genes were differentially expressed, with 13% (799) down-regulated and 8% (534) up-regulated in the Δ*gacS* mutant relative to the wild type (using a cut-off of log2FC > 1.0 and *P*_adj_ < 0.05; Fig. S3; Table S4). This indicates that the loss of GacS significantly impacts a broad spectrum of genes, underscoring its role as a global transcriptional regulator.

The differentially expressed genes were associated with diverse metabolic pathways. For instance, genes involved in co-factor synthesis, nucleoside and nucleotide synthesis, and oxidative stress protection pathways were significantly down-regulated in the Δ*gacS* mutant compared to the wild type (Fig. S4). In contrast, pathways related to amino acid metabolism, aromatic compound degradation, carbohydrate degradation, and RNA polymerase sigma factors were up-regulated (Fig. S4). These findings highlight the pleiotropic nature of GacS, which modulates a wide range of cellular processes.

In *P. protegens* Pf-5, polyurethanases (e.g., *pueA* and *pueB*) and lipases (e.g., *lipA*, *lipB*, and *lipA1*) are known to contribute to PU degradation (17). Interestingly, despite the Δ*gacS* mutant’s enhanced ability to degrade PU, we did not observe up-regulation of these specific genes (Table S5). This suggests that the increased PU degradation in the Δ*gacS* mutant may be driven by alternative mechanisms, such as the up-regulation of other metabolic pathways or compensatory regulatory networks.

### Dysregulation of iron and sulfur homeostasis in the Δ*gacS* mutant of *P. protegens* Pf-5

**Fig 4:**
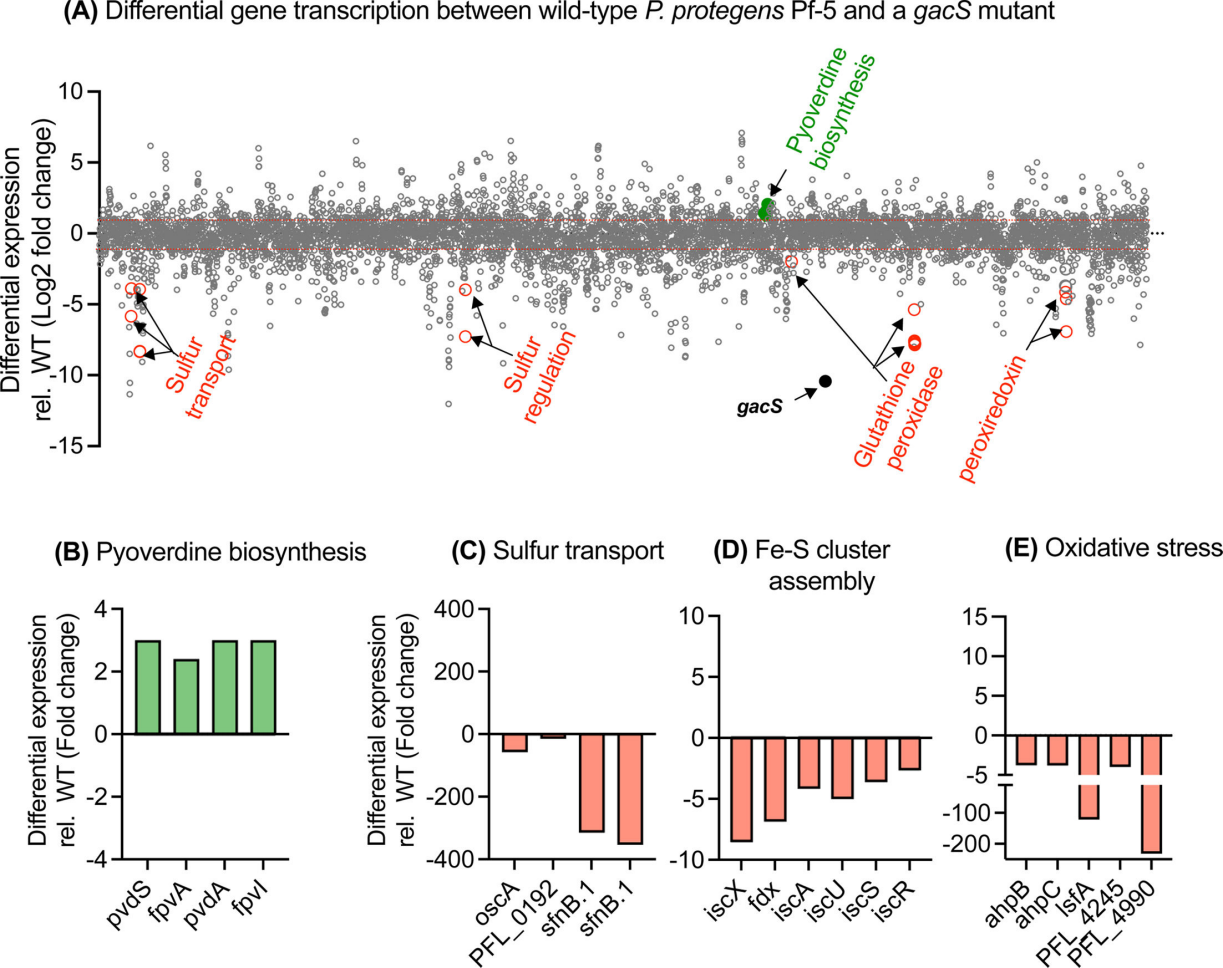
Transcriptomics of *P. protegens* Pf-5 wild-type and its Δ*gacS* mutant. RNA samples were collected for sequencing after 16 h of incubation of bacterial culture in M9 citrate medium with or without Impranil (3 g/L), at 28°C, with orbital shaking at 100 rotations per minute. Bacterial growth was monitored by estimating colony-forming units as shown in Fig. S2. (**A**) Each point represents one of the 6,401 annotated genes in the *P. protegens* Pf-5 genome. The x-axis shows gene order (with the origin of replication at 0 and 6,401), and the y-axis represents the log2 differential expression of each gene in the Δ*gacS* mutant relative to the wild type. The identities of highly modulated, well-characterized gene clusters involved in iron and sulfur acquisition and iron–sulfur cluster biogenesis, and oxidative stress response, are highlighted. (**B–E**) Differential expression of genes related to the pyoverdine biosynthesis (**B**), sulfur transport (**C**), iron–sulfur (Fe–S) cluster assembly (**D**), and oxidative stress protection (**E**).

Iron serves as a vital cofactor in redox-active enzymes for all living organisms (56). Pseudomonas species produce a variety of siderophores, including the pyoverdines (PVDs), to facilitate iron uptake under various iron-limiting environmental conditions (57, 58). Our transcriptomics data showed that four genes associated with PVD biosynthesis were significantly upregulated in the *gacS* mutant of *P. protegens* Pf-5 (Fig. 4A and B). In addition, TonB-dependent outer membrane receptors, a group of proteins that facilitate the uptake of siderophores under iron-limited conditions (59), were also upregulated in the *gacS* mutant (Tables S4 and S6). These results are consistent with results obtained for the *gacA* mutant of *P. protegens* Pf-5 strains (52). Also consistent with the previous study was the upregulation of eight genes encoding RNA polymerase sigma factors. These genes were shown to contain Fur binding sites, suggesting these genes may be regulated by Fur. However, we did not detect any significant change in expression of the *fur* gene itself.

In stark contrast to iron acquisition genes, the inactivation of *gacS* negatively affected genes involved in iron assimilation proteins. For example, the entire gene cluster (*iscRSUAX*) and *fdx* responsible for iron–sulfur (Fe–S) biogenesis and assembly (60, 61) were significantly downregulated up to eightfold (Fig. 4A and D). Fe–S cluster proteins play a critical role in protecting cells from oxidative stress by sequestering intracellular Fe²^+^ ions and storing them as Fe³^+^ oxyhydroxide minerals. This process prevents the generation of harmful hydroxyl radicals through the Fenton reaction (62, 63). We also found genes involved in sulfur starvation response, such as *oscA* (PFL_0191), and sulfur transport genes, including PFL_0192 and PFL_0193, *cysP* (sulfate ABC transporters), and PFL_0244 and PFL_0245 (two SfnB family sulfur acquisition oxidoreductases), were downregulated by up to 300-fold in the *gacS* mutant (Fig. 4C).

### GacS influences genes involved in the oxidative stress response

In addition to the dysregulation of iron and sulfur homeostasis, we also observed downregulation of several genes responsible for oxidative stress responses. For example, in the Δ*gacS* mutant, transcript levels of *ahpB* (PFL_4857) and *ahpC* (PFL_3395), encoding alkyl hydroperoxide reductase subunits B and C, respectively, were significantly downregulated (Fig. 4E). Beyond these alkyl peroxiredoxins, other key enzymes involved in peroxide detoxification, such as 1-cys peroxiredoxin *lsfA* (PFL_5939) and glutathione peroxidases PFL_4245 and PFL_4995, were downregulated by up to 200-fold in the Δ*gacS* mutant. These results suggest that GacS plays a pivotal role in modulating the expression of antioxidant genes, including those involved in Fe–S cluster biogenesis and peroxide detoxification, to ensure cellular survival under oxidative stress conditions.

### Assessment of microbial viability and fitness during growth in PU

To determine if the Pf-5 and Δ*gacS* mutant cells are healthy and fit growing in Impranil over time, we performed a long-term directed evolution assay. We cultured cells for 3 weeks and measured cell viability with plating every 2–3 days. From this, we observed that *P. protegens* Pf-5 wild-type cells were living healthily in PU and maintaining their cell viability levels, but the Δ*gacS* mutants were not (Fig. S5). This further supports that the enhanced ability of the Δ*gacS* mutant to degrade PU is occurring via a distinct mechanism from the wild-type strain. Although the mutant degrades the PU faster than WT, the mutant cells are not healthy over time, possibly due to the increased levels of ROS production.

### Evidence for pyoverdine-mediated Fenton reaction and PU degradation

Iron homeostasis plays a critical role in the oxidative stress response (64). Dysregulation of iron metabolism leads to increased intracellular free iron concentrations (65), which can contribute to the generation of ROS from hydrogen peroxide through the Fenton reaction. Given the observed upregulation of iron acquisition genes and downregulation of hydrogen peroxide scavenging genes like *ahpB*, *ahpC*, *lsfA,* and glutathione peroxidases, we hypothesized that ROS generated via pyoverdine-mediated Fenton reactions might have contributed to the observed increased PU degradation in the Δ*gacS* mutant.

**Fig 5:**
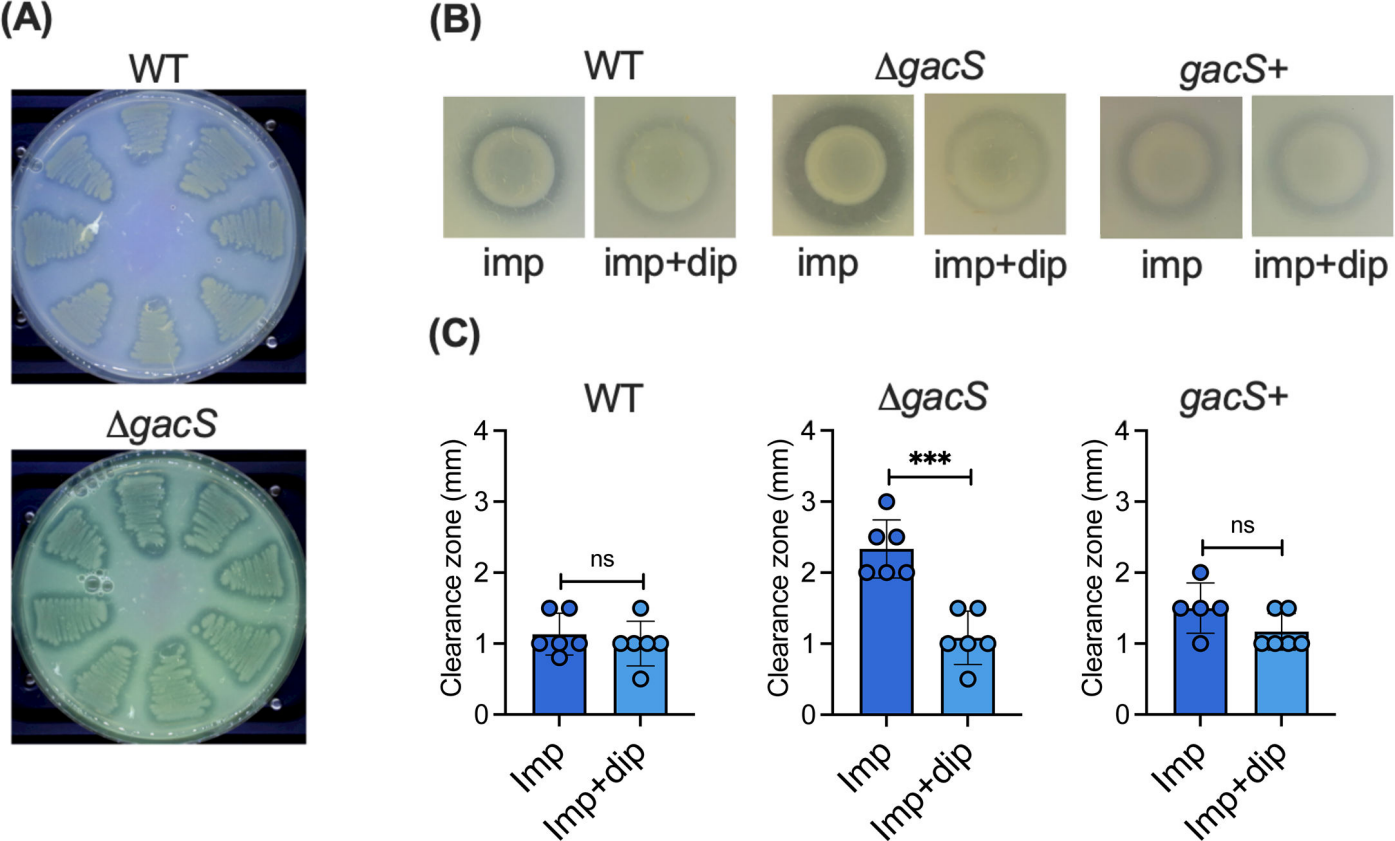
Effect of pyoverdine and the iron chelator dipyridyl on Impranil degradation. (**A**) Eight individual colonies of *P. protegens* Pf-5 wild type (top plate) and its Δ*gacS* (bottom plate) were patched onto M9 citrate + Impranil agar (3 g/L) plates. Green coloration, indicating pyoverdine, was observed on day 4. (**B**) Impranil (imp) clearance by *P. protegens* Pf-5 WT and the Δ*gacS* mutant and *gacS* complemented strain (*gacS*+) with or without added dipyridyl (dip). The final concentration of dipyridyl was 100 µg/mL. Clearing zones, indicating Impranil degradation, were observed on day 4. A representative image from three biological replicates is shown. (**C**) Clearance zone widths were measured by ImageJ and are presented as the means ± standard deviations from three biological replicates. Significance levels were calculated with a two-tailed Student's *t*-test from at least three biological replicates (ns = not significant, ***P < 0.001).

To test this hypothesis, we first assessed pyoverdine production levels in wild-type *P. protegens* Pf-5 and its Δ*gacS* mutant by plating them on agar plates containing citrate and Impranil. As shown in Fig. 5A, a stark green color change in the bacterial patches was observed for the Δ*gacS* mutant, which was not observed for wild-type Pf-5 during PU degradation. This green color change is typical of Pseudomonads producing siderophores, such as pyoverdine, consistent with previously published transcriptomic data showing upregulation of pyoverdine-synthesizing genes in a Δ*gacS* mutant (52) and supporting our hypothesis.

To test whether the pyoverdine-driven Fenton reaction contributes to PU degradation, we used dipyridyl, an iron chelator that restricts iron incorporation into pyoverdine siderophores (66). In the presence of dipyridyl, the wild-type Pf-5 showed little impact on PU degradation (Fig. 5B and C, first panels), whereas the PU clearance ability of the Δ*gacS* mutant was severely impaired (Fig. 5B and C, middle panels). When the Δ*gacS* mutant was complemented with the wild-type *gacS* gene (*gacS*+) using the pBBR1MCS-2 vector, the addition of dipyridyl no longer affected PU clearance (Fig. 5B and C, third panels). These results suggest that the hyperdegrader phenotype of the Δ*gacS* mutant, but not the wild type, is likely linked to pyroverdine production.

**Fig 6:**
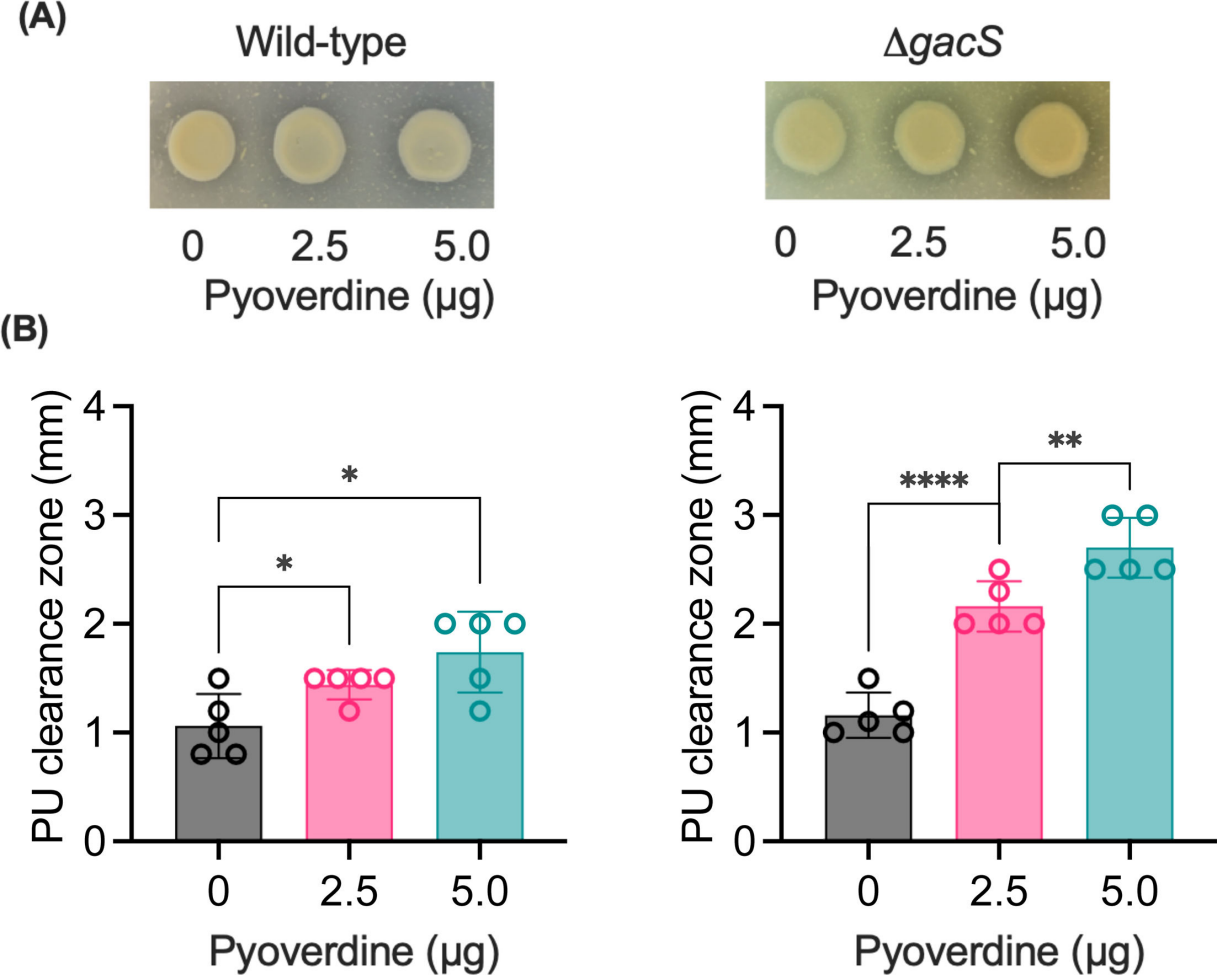
Dose-dependent effect of exogenous pyoverdine on Impranil degradation. (**A**) Wild-type *P. protegens* Pf-5 (left plate) and its Δ*gacS* (right plate) were spotted (10 µL) onto M9 citrate agar plates containing Impranil (3 g/L), and the indicated amount of pyoverdine (Sigma Aldrich) was added. Plates were incubated at 28°C for 72 h. (**B**) The clearance zone widths for the wild-type (left bar graph) and the Δ*gacS* mutant (right bar graph) were quantified by ImageJ and are presented as the means ± standard deviations from at least three biological replicates. Statistical significance was calculated using a two-tailed Student's *t*-test (*P < 0.05, **P < 0.01, ****P < 0.0001).

These findings also raise the possibility of using siderophores themselves as catalysts for bacterial-mediated plastic biodegradation independent of ROS generation. To test this hypothesis, we performed the PU degradation assay by adding pyoverdine exogenously to the bacterial spots on the Imoranil-agar plates. As shown in Fig. 6A and B, in both the wild-type Pf-5 and the Δ*gacS* mutant, the addition of exogenous pyoverdine significantly increased the zone of PU clearance in a dose-dependent manner. However, the extent of the clearance zone was markedly higher in the Δ*gacS* mutant compared to the wild type. These results further indicate that the hyperdegrader phenotype of the Δ*gacS* mutant is likely linked to both increased siderophore production and ROS generation. Due to technical limitations and the physicochemical properties of Avalon, this approach could not be used to validate the inhibitory effect of the *gacS* mutation on Avalon degradation.

Microbial-driven Fenton reactions are known to mediate polystyrene microplastic biodegradation (67, 68). To our knowledge, this study is the first to reveal the molecular details of this process, particularly the role of pyoverdine-driven Fenton reaction in the degradation of non-microplastic polymers, such as PU. However, the Fenton reaction may not be the sole explanation for PU degradation utilized by *P. protegens* Pf-5. This is evident from the observation that complementation of *pueA* in the Δ*pueA* mutant restored Impranil clearance ability independent of the *gacS* mutation.

### Conclusions

Currently, knowledge of PU degradation is primarily focused on genes known to play a role in aiding degradation, particularly secreted enzymes that directly hydrolyze PU bonds. By applying the TraDIS-based TIS functional genomics approach alongside transcriptomics, we were able to gain insight into the regulatory and central metabolic processes involved in PU degradation. This approach allowed us to interrogate the genes and pathways that enhance or hinder bacterial growth on two different PU types. Notably, genes involved in the Mla pathway were found to play a crucial role in fitness on both PUD and TPU.

**Fig 7:**
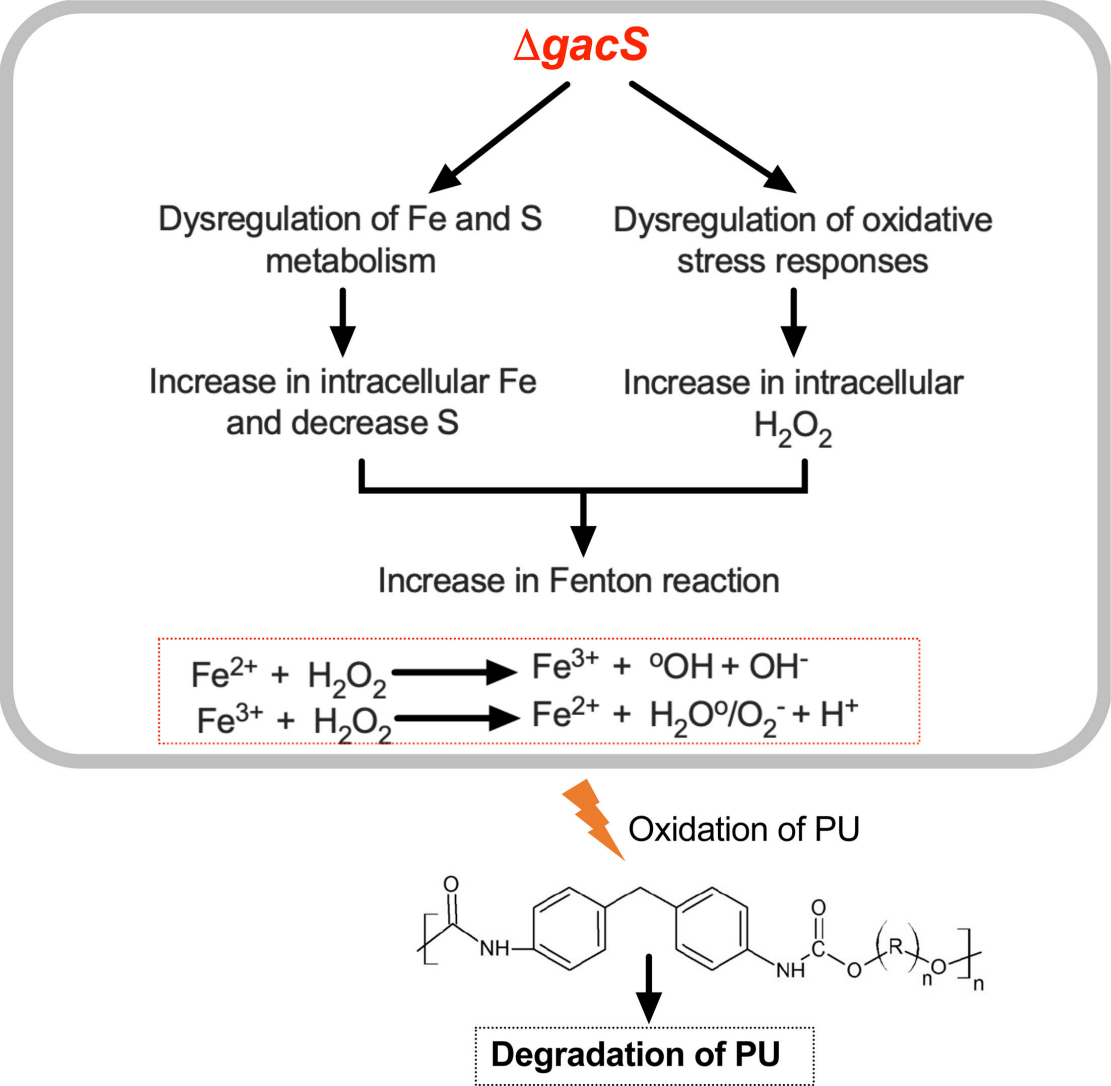
Model depicting the role of GacS in iron homeostasis, oxidative stress response, and Fenton reaction-driven plastic degradation.

Most significantly, this study highlights the critical role of GacS in regulating polyurethane biodegradation in *P. protegens* Pf-5. Transcriptomics analysis revealed that GacS inactivation significantly altered gene expression, impacting diverse metabolic pathways, including iron and sulfur homeostasis, oxidative stress responses, and aromatic compound degradation. Interestingly, the Δ*gacS* mutant exhibited enhanced PU degradation, likely driven by pyoverdine-mediated Fenton reaction rather than the upregulation of known polyurethanases or lipases. The upregulation of siderophore biosynthesis genes and downregulation of antioxidant genes in the Δ*gacS* mutant suggest a mechanism in which increased intracellular iron and ROS contribute to PU breakdown (Fig. 7). Experimental validation using iron chelators further supported this hypothesis, emphasizing the dependence of the Δ*gacS* mutant on siderophore (pyoverdine) activity for its hyperdegrader phenotype.

Additionally, our findings highlight the deleterious effects of the GS, ETC genes on PU degradation, as well as reduced fitness of oxidative stress response genes (e.g., *oxyR* and *ahpC*) in TraDIS analysis. These results further support the role of oxidation processes in PU degradation. Overall, this study provides deeper insights into the genetic and regulatory mechanisms of PU degradation and opens up opportunities to harness bacterial pathways and siderophores for innovative plastic recycling strategies.

## MATERIALS AND METHODS

### Strains, cultivation conditions, and media

For all experiments, we used *P. protegens* Pf-5 wild-type strain, along with its Δ*gacS* and Δ*pueA* CRISPR knock-out mutants. *P. protegens* Pf-5 transposon mutant library generated previously (69) was used in the TraDIS experiments of this study. Strains were streaked onto Luria-Bertani (LB) agar plates from frozen glycerol stocks and incubated at 28°C for 24 h before being used to inoculate into LB broth unless otherwise stated. *E. coli* DH10β cultures (Thermo Fisher Scientific) were grown at 37°C in LB medium. To make PU agar plates, the colloid Impranil DLN was added (at 3 g/L) to M9 medium containing salts (0.2 mM CaCl_2_ and 3 mM MgSO_4_) and 20 mM citrate. Avalon 80 (Huntsman) agar (~3 g/L) was made by melting the PU film in tetrahydrofuran at 60°C for 2 h. After the addition of melted Avalon 80 to molten M9 agar, plates were kept in a fume hood for 24 h before use. Kanamycin (50 µg/mL) and tetracycline (50 µg/mL) were added to the medium when needed.

### Construction of Δ*gacS* and Δ*pueA* mutants

Construction of Δ*gacS* and Δ*pueA* mutants of *P. protegens* Pf-5 was performed using CRISPR-Cas methods as described by Chen et al. (53) with additional modifications. Plasmid vectors pACRISPR and pCasPA were obtained from Addgene (plasmid numbers 113,348 and 113,347). pACRISPR was further modified to remove one of three *Bsa*I restriction enzyme recognition sites, and the ampicillin (*bla*) resistance gene was swapped out with a kanamycin (Km) resistance gene, yielding the modified pACRISPR_KmN vector. PCR was performed using Phusion High-Fidelity DNA Polymerase (Thermo Fisher Scientific). Geneious Prime software (v11.0.15) was used for single-guide RNA primer design (70). Annealed spacer primers were ligated into *Bsa*I restriction enzyme sites of pACRISPR_KmN using T4 DNA ligase (New England Biolabs) and transformed into chemically competent *E. coli* DH10β strains (Thermo Fisher Scientific). NEBuilder HiFi DNA Assembly Master Mix (New England Biolabs) was used to construct the repair template for the gene of interest into the pACRISPR_KmN vector containing the spacer of interest. Primers and plasmids used in this study are listed in Tables S7 and S8, respectively. pCasPA and pACRISPR_KmN, assembled with the spacer and the repair template constructs, were transformed into electrocompetent *P. protegens* Pf-5. Expression of Cas9 and λ-red system was induced by adding L-arabinose (Sigma-Aldrich) to a final concentration of 2 mg/mL. CRISPR mutants were screened via PCR, and the plasmids were cured by growing in 5% sucrose (wt/vol) (Sigma-Aldrich). *P. protegens* Pf-5 mutants were confirmed via PCR and Sanger sequencing (Macrogen Inc., South Korea).

### Impranil and Avalon clearance tests

Overnight cultures of *P. protegens* Pf-5 strains in 3× biological replicates per strain were grown with 200 rpm shaking at 28°C in M9 medium with citrate supplemented at 20 mM. Cells were washed 2× with M9 minimal media containing no carbon source, and 20 µL of each replicate was spotted onto Impranil + citrate or Avalon agar plates. ImageJ (National Institutes of Health) was used to measure the width of clearance zones, where eight distances were used to find the average of the petri plate dimensions in pixels. The average of these was then used to set the pixel aspect ratio. The width of each of the 3× biological replicates was measured in 4× different areas around the colony circumference (Table S3).

### Measuring Avalon 80 (TPU) degradation

For Avalon 80 degradation analysis, the TPU film was melted at 45°C for 4 h, after which 50 µL was drop cast onto sterile glass slides in a sterile petri dish. This was allowed to dry at ambient temperature overnight. *P. protegens* Pf-5 strains were grown as biofilms in M9 medium with citrate at 20 mM and were harvested at day 5 to collect sterile filtrates as described above for the Impranil degradation experiment. One milliliter of sterile filtrate was spotted onto the drop cast TPU film. Plates were then kept at ambient temperature for 9 days, after which the slides were washed 3× with Milli-Q water. Once the slides had dried at ambient temperature, they were examined using FTIR spectroscopy.

### FTIR spectroscopy

FTIR spectra were collected with a Nicolet iS5 FTIR spectrophotometer in transmittance mode using an iD5 diamond ATR accessory (Thermo Scientific), and spectra in the range of 4,000–900 cm^−1^ were recorded. A total of 30 scans were recorded with a resolution of 4 cm^−1^. Background spectra were collected on empty glass slides before analysis. For comparison between samples, spectra were fitted to automatic baseline correction and analyzed using Thermo Fisher Scientific Omnic Software.

### Transposon-directed insertion site sequencing experiment

A 10 µL aliquot of a thawed *P. protegens* Pf-5 TraDIS library carrying approximately 2.5 × 10^5^ unique mutants (69) was inoculated into phosphate-buffered saline to a final concentration of 1.5 × 10^8^ cells. One hundred microliters of the library was spread plated onto M9 medium agar (1.5%) containing either citrate (20 mM) + Impranil (3 g/L) agar, citrate (20 mM) + Avalon 80, or citrate only (20 mM) plates. Each agar type was performed in triplicate. Plates inoculated with the TraDIS library were incubated at 28°C for 48 h. A 10% glycerol was used to gently scrape bacterial lawn off plates. Samples were normalized to 1.6 × 10^9^ cells, and DNA was extracted using the DNeasy extraction kit (Qiagen). TraDIS sequencing was performed at Ramaciotti Center for Genomics (Australia) on a MiSeq to obtain 52-bp single-end genomic DNA reads.

### Bioinformatic analysis

Transposon insertion sites from TraDIS were mapped to the *P. protegens* Pf-5 (CP032358.1) reference genome, and statistical analysis was performed using the TraDIS toolkit (71). Insertions at the last 10% of the 3′ end for each gene were filtered out, as disruptions in this region are likely not able to inactivate the gene. OrthoVenn2 was used to find orthologs between the *P. protegens* Pf-5 (CP032358.1) and the *P. protegens* Pf-5 (CP000076.1) genomes. For those that had no hits with OrthoVenn2, blastn was used to query specific CP032358.1 genes against the CP000076.1 genome, and genes were not annotated if there was no ortholog. The blastn step was done only for genes with significant *P* values ≤0.05 and log2FC ≥1. Genes encoding hypothetical proteins or pseudogenes were classified as putative PU degradation genes. BioCyc (72) and KEGG (73) databases were used to map genes to pathways, and Cytoscape (74) was used for visualizing the *gacS* network.

### Transcriptomic analyses

Three independent *P. protegens Pf-5* and its Δ*gacS* mutant were grown overnight in 5 mL M9 supplemented with 20 mM citrate and 3 g/mL impranil with shaking at 100 rpm at 28°C. RNA extraction was carried out using the miRNeasy mini kit (Qiagen), and DNA was eliminated using the TURBO DNA-free kit (Ambion Inc., USA), as per the manufacturer’s instructions. Libraries were constructed using the Universal Prokaryotic RNA-Seq Library preparation kit (Tecan, USA) according to the manufacturer’s protocol. The samples were sequenced on the Novaseq Illumina platform, producing ~3 million 150 bp paired-end reads per sample and ~25 Gbp of data in total, and analyzed as described previously (75). Reads were quality controlled using FastQC and trimmed using bbduk (v38.79) with the included adapters.fa file and parameters ktrim=r k=23 mink=11 hdist=1 qtrim2=t trimq=10 tpe tbo. Reads were then mapped using bbmap (v38.79) with parameters k=13 and ambig=toss against the *P. protegens* Pf-5 genome (accession: NC_004129.6), sorted using samtools (v1.6), and quantified using HTSeq (v0.12.4) with default parameters. Read counts were aggregated using a custom Perl script and used as the basis for differential expression analysis. Differential expression analysis was performed in the R language, using the edgeR package (v3.30.3) using the quasi-likelihood fit and test functions (glmQLFit, glmQLFTest). Genes differentially expressed, as defined by >2-fold change and *P*_adj_ <0.05, are listed in Table S2.

For visualization of metabolic pathways in our RNA sequencing data, we used BioCyc (76) database in Omics Dashboard Tool for *P. protegens* Pf-5. Enrichment or depletion of metabolic pathways was then analyzed using Fisher’s exact test hypothesis and significant values of <0.05. Enrichment or depletion scores (−log_10_ *P* values) for each pathway in the dashboard were downloaded, and figures were then created using PRISM graphing software (Graph-Pad Software Inc.).

### Construction of *gacS* and *pueA* complementation plasmids

To complement the in-frame deletions, the coding sequences of *pueA* and *gacS* were PCR amplified from the genomic DNA of *P. protegens* Pf-5. The primers were designed to incorporate *Hin*dIII and *Bam*HI restriction sites (Table S7) to facilitate directional cloning into the broad-host-range vector pBBR1MCS-2 (77). This strategy placed each gene under the control of the vector’s lactose-inducible promoter (P*_lac_*). Following digestion and ligation with T4 DNA Ligase, the constructs were transformed into *E. coli* DH10β. The final plasmids, pBBR1MCS-2-*pueA* (7056 bp) and pBBR1MCS-2-*gacS* (8096 bp), were confirmed by Sanger sequencing and subsequently transformed into the corresponding Δ*pueA* and Δ*gacS* mutant strains.

The complementation of *gacS* and *pueA* was investigated by Impranil clearance assays in M9-agar plates with or without the addition of dipyridyl. For all complementation experiments, 0.5 mM isopropyl β-D-1-thiogalactopyranoside was also added to the growth medium.

## Data Availability

Transposon-directed insertion site sequencing (TraDIS) reads were deposited in the Sequence Read Archive (SRA) at the National Center for Biotechnology Information (NCBI) under BioProject PRJNA955671 with BioSample accessions SAMN34179450, SAMN34179451, and SAMN34179452. The RNAseq data have been deposited in the European Nucleotide Archive (ENA) at EMBL-EBI under the project accession number PRJEB86131. All analyzed data sets, along with the primer sets used for mutant construction and genetic complementation, are provided in Tables S1 to S8.

## SUPPLEMENTAL MATERIAL

Supplemental Figures mbio.03544-25-s0001.docx Figures S1-S5.

Supplemental Tables mbio.03544-25-s0002.xlsx Tables S1-S8.

ASM does not own the copyrights to Supplemental Material that may be linked to, or accessed through, an article. The authors have granted ASM a non-exclusive, world-wide license to publish the Supplemental Material files. Please contact the corresponding author directly for reuse.
