# Supplementary material for: Molecular insights into polyurethane biodegradation in Pseudomonas protegens
Source: mBio. 2026 Jan 20;17(2):e03544-25. doi: 10.1128/mbio.03544-25 (PMC12892942; doi:10.1128/mbio.03544-25)
Supplement: Supplemental Figures — Figures S1-S5. [file mbio.03544-25-s0001.docx]

**Supporting Information**

**Molecular insights into polyurethane biodegradation in Pseudomonas protegens**

Lucie Semenec^1*^, Ram Maharjan^1*^, Vaheesan Rajabal^1^, Aidan P Tay, Xin Xu^1^, Hannah Lott^1^, Fiona Facey^1^ Hue Dinh, Sasha G Tetu^1^, Thomas C Williams^1^, Ian T Paulsen^1^, Amy K Cain^1^^

**Authors’ affiliations**

1. ARC Centre of Excellence in Synthetic Biology, Department of Natural Sciences, Macquarie University, Sydney, NSW, 2109, Australia

* Contributed equally

**^^^Corresponding author:** Amy K. Cain [amy.cain@mq.edu.au](mailto:amy.cain@mq.edu.au)

**Running tinning title:** Genetic and Metabolic Insights into PU Biodegradation by *Pseudomonas protegens*

**Key words**

*gacS*, functional genomics, Oxidative stress, Plastic degradation

**Figure S1**

**Figure S1.** Clearance zones corresponding to PU degradation on Impranil (PUD) - and Avolon-citrate plates. Control plates neither contain PUD or Avalon. Clearance zone widths were measured by ImageJ and are presented as the means +/- standard deviations from three biological replicates. Significance levels were calculated with a two-tailed Students t-test from at least three biological replicates (*p < 0.05, **p < 0.01).

**Figure S2.**

**Figure S2. Growth estimation of *Pseudomonas protegens* Pf-5 wild-type (WT) and its Δ*gacS* mutant in M9 citrate with or without added impranil.** *P. protegens* Pf-5 strains in 3x biological replicates per strain were grown for overnight with 100 rpm shaking at 28°C in M9 medium in presence of citrate (20 mM). The overnight grown cultures were then diluted 100-fold in 5 ml M9 citrate (20 mM) without (A) or with Impranil (B) and incubate at 28°C with shaking at 100 rpm. The final concentration of impranil in media was 3g/L. Bacterial growth was monitored by plating appropriate dilutions on LB-agar plates. CFUs were enumerated at various time-points following serial dilution and plating. The data are a representative of three replicate experiments. Blue arrow indicates the time point that samples were collected for RNA extractions.

**Figure S3**

**Figure S3.** **Volcano plot showing differential gene expression between wild-type and the gacS experimental conditions.** The volcano plot displays the log₂ fold change (x-axis) versus the –log₁₀ adjusted *p*-value (y-axis) for all genes analysed. Each dot represents an individual gene. Genes that are significantly upregulated (log₂FC > 1, padj < 0.05) are shown in green, and significantly downregulated genes (log₂FC < –1, padj < 0.05) are shown in red. Grey dots represent genes that did not show significant differential expression. The horizontal dashed line indicates the adjusted *p*-value threshold of 0.05, and the vertical dashed lines mark the fold change cut-offs (±1 log₂FC).

**Figure S4**

**Figure S4.** **Pathway enrichment analysis of significantly changing genes (log2FC >1.0 change and Padj<0.05) between Pseudomonas Protegens Pf-5 wild-type (WT) and its Δ*gacS* mutant.** To visualise metabolic pathways in our RNA sequencing data, we used the BioCyc database within the Omics Dashboard Tool. Information in Table S3 was imported into BioCyc (Biocyc.org) and analysed using the Omics Dashboard. Enrichment (upregulation) or depletion (downregulation) of metabolic pathways then analysed using the Fisher’s exact test hypothesis and significant values of <0.05. Enrichment or depletion scores (-log_10_ P values) for each pathway in the dashboard were downloaded, and figures were then created using PRISM graphing software (Graph-Pad Software Inc).

**Figure S5**

**Figure S5.** **Assessment of microbial viability and fitness during growth in PU.** Survival of *Pseudomonas protegens* Pf-5 wild-type (WT) and its Δ*gacS* mutant in M9 citrate-impranil culture was followed by estimating colony forming units (CFU) over indicated time points. Approximately 10^5^ cells from an overnight culture of WT and its Δ*gacS* mutant was sub-cultured into 5 ml M9 citrate medium containing 3 g/l impranil and incubated at 28°C with shaking 100 rpm. Samples were taken at indicated time points and 10-fold serially diluted in sterile PBS and 10 µl of each dilution was then spotted on Mueller Hinton-agar plates. Plates were incubated at 28°C 48 hours and colonies were enumerated to determine the surviving cells as CFU/ml. Error bars represent standard deviations from three independent biological replicates.
